# Neoadjuvant Chemotherapy for Breast Cancer: Evolution of Clinical Practice in a French Cancer Center Over 16 Years and Pathologic Response Rates According to Tumor Subtypes and Clinical Tumor Size: Retrospective Cohort Study

**DOI:** 10.26502/jsr.10020251

**Published:** 2022-09-14

**Authors:** Gilles Houvenaeghel, Alexandre de Nonneville, Monique Cohen, Frédéric Viret, Sandrine Rua, Laura Sabiani, Max Buttarelli, Emmanuelle Charaffe, Audrey Monneur, Aurélie Jalaguier-Coudray, Marie Bannier, Renaud Sabatier, Anthony Gonçalves

**Affiliations:** 1 Aix-Marseille University, CNRS (National Center of Scientific Research), INSERM (National Institute of Health and Medical Research), Paoli-Calmettes Institute, Department of Surgical Oncology, CRCM (Research Cancer Centre of Marseille), 13009 Marseille, France; 2 Aix-Marseille University, CNRS (National Center of Scientific Research), INSERM (National Institute of Health and Medical Research), Paoli-Calmettes Institute, Department of Medical Oncology, CRCM (Research Cancer Centre of Marseille), 13009 Marseille, France; 3 Aix-Marseille University, CNRS (National Center of Scientific Research), INSERM (National Institute of Health and Medical Research), Paoli-Calmettes Institute, Department of Pathology, CRCM (Research Cancer Centre of Marseille), 13009 Marseille, France; 4 Aix-Marseille University, CNRS (National Center of Scientific Research), INSERM (National Institute of Health and Medical Research), Paoli-Calmettes Institute, Department of Radiology, CRCM (Research Cancer Centre of Marseille), 13009 Marseille, France

**Keywords:** Breast cancer, Neoadjuvant chemotherapy, Clinical practice, Pathologic response

## Abstract

We examined characteristics trends in early breast cancer patients receiving neoadjuvant chemotherapy (NAC) over a 16-year period. Our primary objective was to analyze variations in tumor stage and subtype over time. Secondary objectives included analyses of type of surgery and pathological response, from January 2005 to May 2021, 1623 patients receiving NAC were identified. Three periods were determined: 2005-2009 (P1), 2010-2014 (P2), 2015-2021 (P3). Correlations between periods and patient features with cT stage, pathological breast and axillary node response, pathological complete response (pCR), and type of surgery were assessed in univariate and multivariate analyses. We observed a significant increase in cT0-1 and N0 stages with periods (from 6.8% at P1 to 21.2% at P3, and from 43.2% at P1 to 55.9% at P3, respectively) and in the proportion of HER2+ and triple negative (TN) subtypes. In a multivariate analysis, a decrease of cT2-3-4 tumors during P3 was observed for HER2+ (OR:0.174; p=0.004) and TN tumors (OR:0.287; p=0.042). In-breast pCR and pCR were observed in 40.8% and 34.4% of all patients, respectively, with strong association with tumor subtypes, but not with tumor size in multivariate analysis (37.0% pCR for cT0-1 tumors, 36.4% for cT2 tumors, 29.1% for cT3 tumors (cT0-1 versus cT≥2; p=0.222)). pCR was negatively associated with cN1 stage (OR:1.499; p<0.001 for cN1 patients compared to cN0). We observed an increase in the proportion of small cT0-1 and N0 stages treated with NAC, especially in HER2+ and TN subtypes. No significant impact of tumor size on pCR rates was found.

## 1. Introduction

Neoadjuvant chemotherapy (NAC), as defined as the administration of cytotoxic treatment before tumor surgical removal, has been implemented in breast cancer (BC) during the last 50 years, encompassing different objectives. Initially dedicated to inoperable BC to convert it into a surgically removable disease [1,2], NAC was then widely developed in operable BC when frontline breast-conserving surgery with “in sano” margins was not feasible [3], based on accumulating data showing a high rate of clinical and pathologic response (pCR) as well as identical survival outcome when compared to adjuvant chemotherapy [4]. Yet, the development of more and more sophisticated oncoplastic techniques could also increase the possibilities of immediate breast-conserving surgery [5,6]. Even though some initial expectations in favor of NAC were not confirmed, including a potential survival advantage over adjuvant chemotherapy by an early impact on the hypothetic surgical stimulation of micro-metastatic disease and tumor shedding [7], the interest in NAC has been further boosted during the last decade in relation with several observations. First, molecular analysis of BC revealed that the disease was composed of different subtypes with distinct survival outcomes [8,9], including HER2-positive and triple-negative BC, two subtypes in which both survival benefits from adjuvant chemotherapy and probability of reaching pCR after NAC are the highest. Second, a tight relationship was demonstrated between the achievement of pCR and survival, most notably in the latter subtypes [10–12]. Third, recent randomized trials found that in those patients with residual invasive disease after preoperative systemic treatment, adjuvant trastuzumab emtansine in HER2-positive BC treated with trastuzumab-based NAC [13] and capecitabine in triple negative (TN) BC treated with anthracycline-taxane NAC [14] both significantly improved survival. These recent practice-changing results have resulted in making NAC a standard of care in most of these BC subtypes, even when initial breast conservation may be achieved by frontline surgery. In this retrospective monocentric study, we have examined how the use of NAC has evolved during the last 16 years in a French comprehensive cancer center, including clinical and pathological features of treated patients, as well as pathologic complete response (pCR) results. The primary objective was to analyze variations in tumor stage and subtype over time. Secondary objectives included analyses of the type of breast and axillary surgery as well as pathological axillary and in-breast response.

## 2. Materials and Methods

### 2.1. Patient selection and study design

**Figure 1:**
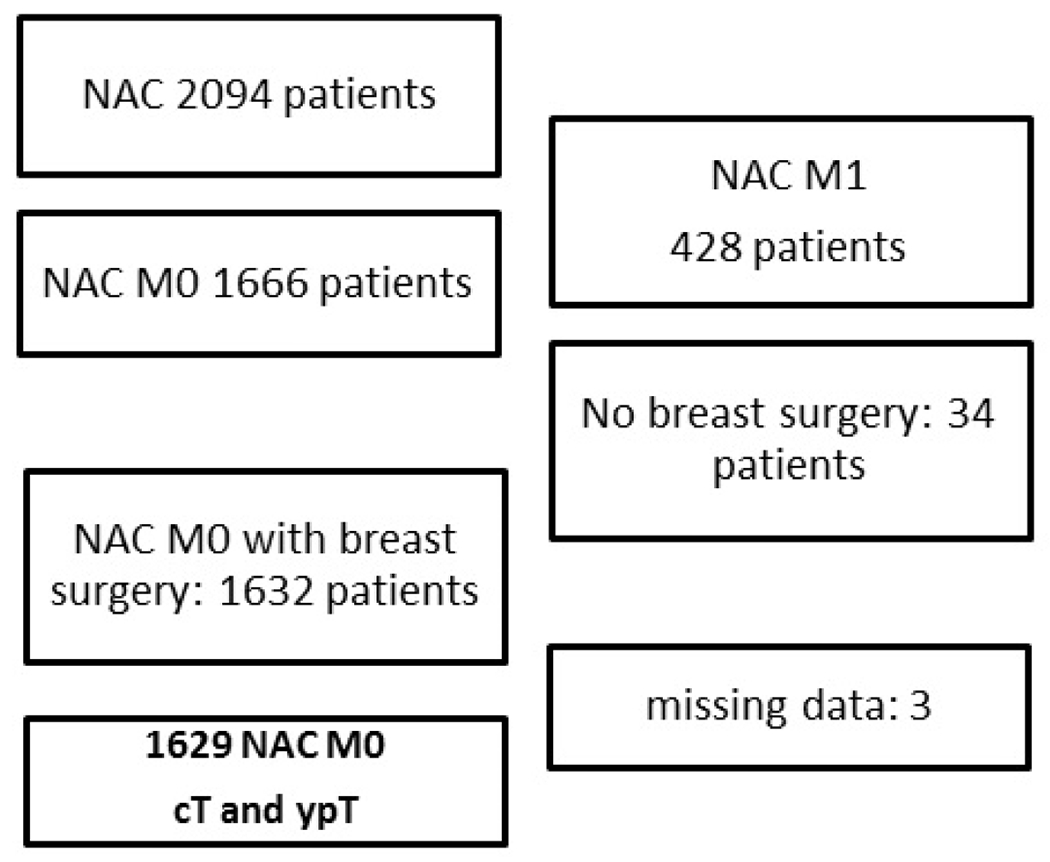
Flow chart. Abbrevations: NAC: neoadjuvant chemotherapy; MO, without synchronous distant metastases; cT, clinical tumour size stage; ypT, pathologic tumour status after neoadjuvant chemotherapy

Medical records of early BC patients treated from January 2005 to May 2021 were retrieved from our institutional clinical databases for retrospective analysis. This cohort study was approved by our institutional review board (Registered study in Clinical research Institute: NAC-TS-IPC 2021-026). Patient and tumor characteristics, periods, treatments, and pathological results were collected. We included in the present study 1623 patients treated with NAC, without metastasis at initial diagnosis (figure 1). Patients receiving NAC were staged using clinical examination, mammography and ultrasonogaphy, breast MRI. Search for distant metastases using either PET-scan or a combination of CT-scan and bone scan. Evaluation of lymph node status was determined by sentinel lymph node biopsy (SLNB) with or without completion axillary lymph node dissection (ALND) or only ALND. SLNB was realized before NAC for cN0 patients with cT ≤ 5 centimeters. The method used for the detection of SN was a combined technique or isotopic only detection during the last years. NAC included anthracyclines plus taxanes-based regimen. All patients with HER2+ disease received trastuzumab during NAC. Patients who had residual components of ductal carcinoma in situ were assessed as having a breast pathological complete response based on the National Surgical Adjuvant Breast and Bowel Project criteria [15]. Patients with axillary residual lymph node tumor (ypN1) were assessed as having no pathological complete response. Pathological complete response (pCR) was defined as [ypT0 or ypTis] and [ypN0 or pN0sn or pN1sn without ALND or ypNx (absence of axillary surgery)] [16]. Endocrine Receptor (ER) and HER2 status were determined according to French guidelines (estrogen and/or progesterone receptors by IHC with a 10% threshold for endocrine receptor (ER) positivity; IHC HER2-positivity score of 3+ and/or HER2 amplification by in situ hybridization) [17,18]. Five IHC tumor subtypes were defined as surrogates for molecular subtypes based on tumor grade, ER and HER2 status: luminal A-like (ER+/HER2−/grade1 or 2), luminal B-like (ER+/HER2−/grade 3), luminal B-like HER2+, HER2+ (ER−/HER2+), and triple-negative (ER−/HER2−) [16]. The work has been reported in line with the STROCSS criteria [17].

### 2.2. Statistical Analysis

Descriptive statistics were used to describe the categorical (counts and frequencies) and continuous (median and range) variables. Characteristics of patients were compared by different periods: Period 1 (P1) 2005-2009, period 2 (P2) 2010-2014, and period 3 (P3) 2015-2021 by using χ2 test for categorical variables, and Kruskal-Wallis test for continuous variables. The main characteristics of patients and tumors were categorized (cT0-1 stage vs. cT2-3-4, ypT0-is vs. ypT≥1, pCR vs. no pCR, breast conservative surgery vs. mastectomy) and association with other variables were explored in univariate, and multivariate analysis by binary logistic regression for significant criteria in univariate analysis. Statistical significance was set as p ≤ 0.05. Analyses were performed with SPSS version 16.0 (SPSS Inc., Chicago, Illinois).

## 3. Results

### 3.1. Characteristics of patients according to periods

Of 1629 patients included, 280 received NAC during P1, 503 during P2, and 846 during P3. Median age for all patients was 50.0 years and was stable among periods. Characteristics of patients are reported in table 1. All the other assessed criteria were significantly associated with periods of treatment. Notably, we observed a significant increase in the proportion of cT0-1 (6.8% at P1 to 21.2% at P3), and node-negative tumors (43.2% at P1 to 55.9% at P3) with periods. The rate of breast conservative surgery increased (36.8% at P1 to 48.5% at P3), whereas ALND decreased significantly with time (74.3% at P1 to 57.7% at P3). Similarly, a decrease in the proportion of Luminal-A (36.1% at P1, 27.4% at P2, and 18.2% at P3), and Luminal-B HER2− (19.3%, 14.9%, and 8.2%) tumors was observed, while HER2+ (15.0%, 16.3%, and 21.0%), Luminal B HER2+ (10.7%, 12.5%, and 15.0%), and triple-negatives (18.6%, 28.6%, and 37.5%) increased. Of note, the rate of residual invasive disease in breast and nodes also significantly decreased across the three periods (Table 1).

### 3.2. Axillary surgery details for all patients and for clinically N0 patients

For all patients, axillary surgery type (SLNB, ALND, or SLNB+ALND) was significantly associated with periods, tumor subtypes, cT, and cN stages (Table 2). Considering only cN0 patients, SLNB was performed in 496 cases with completion ALND in 168 patients (33.9%). In cN0 patients, SLNB was significantly associated with the period of treatment (42.1% (51/121) during P1, 71.9% (141/196) during P2, and 64.2% (304/473) during P3 ; p<0.0001), tumor subtypes (49.2% (89/181) in Luminal-A tumors, 63.6% (49/77) in Luminal-B HER2−, 65.7% (94/143) in Luminal B HER2+, 56.0% (60/107) in HER2+, and 72.3% (204/282) in TN ; p<0.0001), and cT stages (63.3% (95/150) for cT0-1 tumors, 71.7% (362/505) for cT2, and 31.7% (38/120) for cT3 ; p<0.0001). In multivariate analysis, SLNB was less frequently performed for cT3 and cT4 tumors compared to cT0-1, but more commonly used for TN tumors (OR: 2.332, CI95% 1.511-3.597 ; p<0.0001), and during P2 and P3 periods (OR: 3.567, CI95% 2.120-6.001; p<0.0001 and OR: 1.877, CI95% 1.181-2.983 ; p=0.008, respectively) (Table 3, SLNB versus ALND section).

### 3.3. cT Stages according to periods

cT stage (cT0-1 versus cT2-3-4) was significantly associated with periods, and cN stages, while the proportion of different subtypes did not differ significantly by univariate analysis (Table 4). The multivariate analysis revealed a strong association of cT2-3-4 tumors with cN≥1 (OR: 1.748; p<0.0001), and a notable decrease of cT2-3-4 stages during the third period (OR: 0.270 ; p<0.0001) (Table 3, cT2-3-4 versus cT0-1 section). During the third period, cT0-1 rates increased significantly during years 2018-2021 (122/515: 23.7%), in comparison with years 2015-2017: (57/331: 17.2%); p=0.015) for all tumor subtypes, and for HER2-positive/ER-negative (21.4%: 18/84 versus 14.0%: 6/43), Luminal B HER2-positive (25.4% (31/122) versus 17.9% (10/56)), triple-negative tumors (22.5% (43/191) versus 15.1% (19/126)) [23.2% (92/397) versus 15.6% (35/225); p=0.014, considering together these three last subtypes]. There was no significant difference observed in Luminal A and Luminal B HER2-negative tumors: 25.4% (30/118) during years 2018-2021 versus 20.0% (21/105) during years 2015-2017 (p=0.211). Tumor subtypes in depth analysis showed higher rates of cT0-1 tumors during the third period for all tumor subtypes, except for Luminal B HER2-negative tumors (Table S1). In a multivariate analysis adjusted on periods and cN status, a decrease of cT2-3-4 tumors during the third period was observed for Luminal A tumors (OR: 0.197, CI 95% 0.079-0.489; p<0.0001) with an increase of cN1 patients (OR: 1.927, CI 95% 1.072-3.465; p=0.028), for HER2+ (Luminal B HER2+ & HER2+) (OR: 0.174, CI 95% 0.053-0.573; p=0.004) without significant difference for cN status (OR: 1.454, CI 95% 0.865-2.445; p=0.158), for triple-negative tumors (OR:0.287, CI 95% 0.086-0.956; p=0.042) with an increase of cN1 patients (OR: 1.814, CI 95% 1.073-3.066; p=0.026) and without significant difference for Luminal B HER2− tumors (OR: 0.616, CI 95% 0.215-1.768; p=0.368, and OR: 2.067, CI 95% 0.904-4.724; p=0.085, for P3 and cN1 status, respectively).

### 3.4. Pathological results: in-breast tumor response

**Figure 2:**
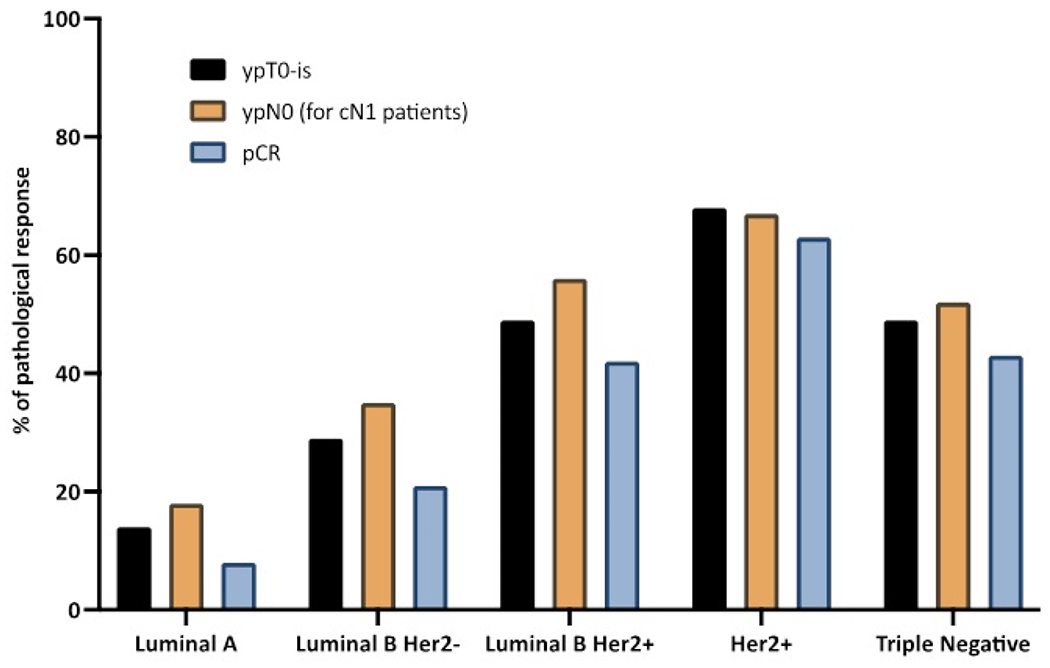
pathological breast tumor response, axillary lymph node response, and pathologic complete responses rates after neoadjuvant chemotherapy. Abbrevations: ypT, pathologic tumor status after neoadjuvant chemotherapy; ypN pathologic lymph node status afer neoadjuvant chemotherapy; pCR, pathologic complete response.

An in-breast pathological complete response ypT0-is was reported in 40.8% of all patients, including 9.0% of in-situ residual tumors (ypTis). A trend toward a lower rate of invasive residual tumor was observed in the third period (480/836: 49.7% for P3 versus 485/783: 61.9% for P1-2). For all patients, a significant association between in breast pathological response (ypT0-is versus ypT≥1) and tumor subtypes was observed in univariate analysis (Table 5): 14.2% for Luminal A (56/393), 28.8% for Luminal B HER2-negative (57/198), 49.0% for Luminal B HER2-positive (148/302), 67.7% for HER2-positive/ER-negative (149/220), and 49.1% for triple-negative tumors (252/513) (Figure 2). In patients with Luminal-A tumors, ypT0-is response rates were 2.8% (2/71) and 16.8% (54/322) for grade 1 and grade 2 tumors, respectively. For Luminal-A grade 2 tumors, ypT0-is response rates were 8.9% (11/124), 15.4% (6/39), and 23.3% (37/159) for tumors with Ki67≤20%, >20%, and unknown, respectively (p=0.005). In multivariate analysis, a strong association was observed between ypT0-is and tumor subtypes, but not with tumor size (Table 6) which was distributed as follows: 45.4% (109/240) of ypT0-is for cT0-1, 41.7% (390/935) for cT2 and 37.1% (130/350) for cT3 tumors (cT0-1 versus cT>1: p=0.077).

### 3.5. Pathological results: axillary lymph node response

Considering cN0 patients, sentinel nodes were involved (pN1sn) in 16.7% (55/330) of cases, and 70.2% (315/449) were ypN0, whereas cN1 patients were ypN0 in 43.8% (344/786) (Table 7). Residual nodal tumor (ypN1) was present in 20.8% (103/496) of ypT0-is tumors and in 63.8% (487/763) of ypT≥1 tumors (p<0.0001), in 6.6% (11/167) and 43.5% (123/283) of ypT0-is and ypT>1 tumors, respectively for cN0 patients, 28.0% (90/322) and 75.8% (354/467) of ypT0-is and ypT>1 tumors, respectively for cN1 patients (20 patients cN unknown) (p<0.0001) (Table 7); in 50.0% (26/52), 30.8% (16/52), 17.2% (20/116), 9.6% (11/115), and 18.2% (29/159) of ypT0-is tumors for Luminal A, Luminal B HER2-negative, Luminal B HER2-positive, HER2-positive/ER-negative and triple negative tumors, respectively (p<0.0001) (Table S2) (Figure 2).

### 3.6. Pathological results: pathological complete response

Pathological complete response rate was reported in 34.4% of all patients, without correlation with clinical tumor size: 37.0% (89/240) pCR for cT0-1 tumors, 36.4% (340/935) for cT2 tumors and 29.1% (102/350) for cT3 tumors (cT0-1 versus cT≥1 ; p=0.222). In univariate analysis, pCR was significantly correlated with tumor subtypes (7.6%, 20.7%, 42.4%, 62.7% and 43.5% for Luminal A, Luminal B HER2−, Luminal B HER2+, HER2+ and triple-negative tumors, respectively; p<0.0001) (Figure 2), cN stage (39.4% and 29.7% for cN0 and cN1, respectively; p<0.0001) and periods (33.6%, 29.6%, 37.6% for P1, P2 and P3, respectively; p=0.011) (Table 5). In multivariate analysis, a strong association was observed between pCR and tumor subtypes, and between pCR and cN1 stage, with more patients with residual breast or nodal tumor for cN1 patients (OR: 1.499, p<0.0001) (Table 6).

### 3.7. Breast conservative surgery or mastectomy

Breast surgery type was significantly associated with periods, tumor subtypes, cT stages, and cN stages: 36.8% (103/280) of breast conservative surgery during P1, 43.7% (220/503) during P2 and 48.5% (410/846) during P3; 38.4% (151/393) of breast conservative surgery for Luminal-A tumors, 45.4% (90/198) for Luminal-B HER2−, 42.7% (129/302) for Luminal-B HER2+, 34.5% (76/220) for HER2+, and 55.5% (285/513) for triple-negative; 52.9% (127/240) for cT0-1 tumors, 54.4% (509/935) for cT2, and 24.3% (85/350) for cT3 (Table 8). In multi-variate analysis, lower rates of mastectomies were observed for triple-negative tumors (OR: 0.567; p<0.0001), whereas mastectomies rates were higher for cT3, cT4 tumors, and cN1 stages (Table 3). There was no significant difference according to periods of treatment.

## 4. Discussion

In this large single-center retrospective study covering a broad 16-year period, we reported an increase in NAC use in successive years for small (cT0-1) tumors, and an increase in the use of SLNB before NAC with no significant difference in pCR and mastectomy rates. In locally advanced and large breast cancers, particularly when mastectomy is required, NAC is recommended to decrease mastectomy rate [19]. In operable patients, NAC has not demonstrated impact on survival in comparison with adjuvant chemotherapy after breast surgery [7,19]. However, NAC allows assessment of pathological response, which has prognostic value, most notably in HER2+ and triple-negative subtypes and can guide the indication of postoperative treatments with a significant reduction in the risk of recurrence, as demonstrated with trastuzumab emtansine and capecitabine in these subtypes, respectively. Consequently, NAC should be preferred for triple-negative and HER2-positive tumors > 2cm [19]. However, NAC could also be discussed for smaller triple-negative and HER2-positive, cT1c, and sometimes cT1b tumors, especially for cN1 or pN1sn patients when SLNB is performed before NAC. Indeed, in HER2+ BC, trastuzumab-based adjuvant chemotherapy has demonstrated robust survival benefits in randomized trials in patients with pT1c stage or higher [20–23]. In addition, accumulating data suggest similar benefits even in sub-centimetric tumors, more convincingly in pT1bN0 than pT1aN0 tumors [24]. Of note, the KATHERINE study [13], in which post-operative trastuzumab emtansine was administered in the presence of invasive residual disease after trastuzumab-based NAC, demonstrated a significant improvement in invasive recurrence, 12% of patients had T1 tumors and there was no evidence in favor of a lower benefit compare to higher tumor stage (HR=0.33 [95% CI 0.13-0.88], HR=0.52 [95%CI 0.35-0.78] and HR=0.38 [95%CI 0.23-0.63]) in T1, T2, and T3, respectively). Thus, since trastuzumab-based chemotherapy provides survival advantages and is commonly recommended even in small tumors, and since the absence of pCR may allow an effective rescue, it is tempting to consider a novel paradigm in which virtually any HER2+ tumors > 5mm may be offered NAC. However, during the same period, a relative de-escalation was proposed to T1-T2 (up to 3cm) node-negative HER2-positive BC patients. Thus, in this patient population, a short-duration (12 weeks) anthracycline- and alkylating-free regimen, using weekly paclitaxel in combination with trastuzumab may be proposed with minimal toxicity [25]. Whether such a regimen could similarly be used in the neoadjuvant setting with a trastuzumab emtansine-based rescue strategy warrants further investigation. Similarly, in triple-negative BC patients, adjuvant chemotherapy is largely recommended whatever tumor size (NCCN, ESMO, St-Gallen), even though survival benefit in pT1abN0 remains unclear [26,27]. Once again, in this subtype, adjuvant capecitabine, in patients receiving neoadjuvant anthracycline +/− taxane-based chemotherapy and with the persistence of residual invasive cancer on surgical specimen has been demonstrated to improve survival, according to CREATE-X phase III trial [14]. Of note, in this study, only 14% of patients had initial cT1 stage but in this subgroup, the HR was in favor of capecitabine (HR=0.65 [95%CI 0.30-1.44]) and was similar to the one observed in tumor larger than cT1(HR=0.71 [95%CI 0.53-0.96]), even though it did not reach statistical significance, presumably due to limited sample size. Conflicting results have been reported when considering adjuvant capecitabine in BC patients and most studies have failed to identify significant survival benefits [13,20–23,28–35]. However, subgroup analyses of some individual studies [28] and recently reported meta-analyses have suggested significant improvements in triple-negative BC patients, most of benefits being driven by studies in which NAC was used to select patients with the highest risk [36,37]. Accordingly, adjuvant capecitabine in patients not reaching pCR after NAC has become standard of care in this subtype. Thus, neoadjuvant anthracycline-taxane chemotherapy could be proposed to any pT1b-c triple-negative breast cancer to better define the need for additional cytotoxic such as capecitabine. Recent results of the OLYMPIA trial [38], in which olaparib given to patients with germline BRCA mutation and BC with invasive residual disease (any invasive in triple-negative BC, high CPS+EG score in ER+ BC) after neoadjuvant treatment, was shown to improve significantly survival may further support this approach in this specific subgroup of patients. The rate of conservative surgery appears to be stable over time, with higher rates for triple-negative tumors. However, the rate of conservative surgery depends on many factors, including the initial tumor size, as we have observed for cT3 and cT4 tumors, and on the patient’s choice of whether or not to have a total mastectomy in the presence of hereditary risk or mutation, when conservative surgery is feasible. This choice may also have evolved with the increased possibilities and proposals for immediate reconstruction. However, patients must be informed of the aesthetic limitations of immediate or delayed reconstruction [39], postoperative complications [40], and of the risks of altering the aesthetic results in the event of post-mastectomy radiotherapy. As expected, pCR rates following NAC were significantly different according to tumor subtypes, being higher in HER2+ and triple-negative BC. Logically, the use of NAC is currently predominantly dedicated to these subtypes. Importantly, no significant differences in pCR according to tumor size was observed, which also argues in favor of considering NAC even in smaller tumors, as discussed above. There was a trend for a slightly higher pCR in the last period, which did not reach statistical significance. This may be due to the lack of significant change in NAC regimen during the considered periods, most HER2+ patients having access to trastuzumab, while pertuzumab or platinum were not routinely used in France in HER2+ and triple-negative subtypes, respectively. Axillary exploration and treatment have evolved significantly in recent years. The performance of an SLNB for cN0 patients (clinical and ultrasound) can be considered before or after NAC [41–43]. SLNB prior NAC for tumors ≤5cm in patients without clinical axillary invasion (cN0) is performed under the same conditions as surgeries performed at the outset. This approach has been proposed for many years [44] and validated more recently by the SENTINA study [41]. In these situations, when the sentinel lymph nodes are not involved, the omission of an additional ALND has been recommended since the results of the NSABP B-32 trial, which validated the sentinel lymph node technique without dissection for immediate surgery [45], and since the results of the SENTINA trial, which included an axillary dissection prior to NAC in one of the randomized arms [41]. In case of sentinel lymph node involvement, completion of ALND after NAC is the attitude that has been usually advocated. However, since the results of the ACOSOG Z0011 and IBCSG 23-01 trials, the omission of completion ALND after NAC can be considered as it is the case for immediate BC surgery, in line with the criteria corresponding to the situations of these trials: presence of at most two macro- or mi-cro-metastases in the sentinel lymph nodes without macroscopic capsular rupture with the realization of breast-conserving surgery, whole-breast radiotherapy, and systemic treatment. Under these conditions, the number of cases with more than 2 sentinel nodes affected being low, an omission of completion ALND would be possible for the vast majority of patients. The presence of micro-metastases or isolated cells is considered to have a prognosis equivalent to pN0sn [19] in case of ER+ tumor [46] but with an unfavorable prognostic value for triple-negative cancers [46]. The disadvantage of this approach is the need for additional surgery, which should not delay the initiation of NAC. On the other hand, the advantage is to precisely know the lymph node stage, in particular to evaluate the indication of regional lymph node radiotherapy, at least in case of macro-metastasis of the sentinel lymph nodes. However, a significant limitation of omitting ALND after initial positive SLND includes the lack of nodal staging allowing to certify pCR status according to the most consensual definition. Yet, in HER2+ and triple-negative BC, a residual nodal involvement when breast pCR is achieved is expected to be uncommon (9.6 to 18.4% in our study). However, the most common approach proposed for cN0 patients, both clinically and ultrasonographically, is to perform an SLNB after NAC [19]. The downstaging linked to the sterilization of initially affected lymph nodes allows considering the omission of an additional ALND in a significant proportion of patients. Conversely, in the case of an invaded node after NAC, a factor associated with poor prognosis, additional ALND is indicated for micro or macro-metastases. The advantage of this approach is to determine the pathologic response of the lymph nodes to NAC. Another advantage is to offer ALND only to a small number of patients, which is probably not higher than the rate of abstention from dissection if SLNB is performed before NAC and without dissection for invaded pN0sn and <2 SN. The disadvantage is that the initial lymph node status is not known, which may be the only element determining the indication or not of regional lymph node radiotherapy. In patients with initial limited axillary lymph node involvement confirmed on needle aspiration or biopsy (cN1), SLNB may be considered in selected cases with clinical response in the axillary region, based on the results of the SENTINA and ACOSOG Z1071 trials [41,48]. However, the rate of false negatives remains high overall, from 8% to 14.2 [41,48,49]. This false-negative rate can be reduced by marking the biopsied lymph node (clip or other technique) and by sampling more than 2 sentinel lymph nodes whenever this number is reached by identification [50,51]. In the meta-analysis of Samiei et al. [52] the axillary pCR rate in cN1 patients pathologically proven cN-positive disease was 60% for HER2-positive/ER-negative tumors, 45% for HER2-positive/ER-positive tumors, 48% for triple-negative tumors, and 18% for HER2-negative/ER-positive tumors. These rates were comparable in our study for cN1 patients, 67.3% (74/110) for HER2-positive/ER-negative tumors, 56.1% (87/155) for HER2-positive/ER-positive tumors, 51.7% (104/201) for triple-negative tumors, 35.3% (41/116) for HER2-negative/ER-positive/Grade 3 tumors, and 18.4% (37/201) for HER2-negative/ER-positive/Grade 1-2 tumors. Although our study includes a considerable number of early BC patients receiving NAC on a broad 16-year period, limitations must be addressed. First, potential selection bias and lack of standardization in treatment strategies inherent in the retrospective design of the study. Second, we chose to include patients until May 2021 and thus, assessment of independent causality between pathologic response and outcome was not possible. Third, no specialized pathology review of all cases was undertaken.

## 5. Conclusions

The pCR rate after NAC was neither higher nor significantly different for tumors <2cm compared with tumors ≥2cm, especially for HER2+ and TN subtypes for which adjuvant therapy can be offered to improve prognosis. The pCR rate appears to correlate with intrinsic tumor characteristics and clinical lymph node status rather than with tumor size. These results suggest that it is possible to propose NAC in patients with these tumor subtypes, in case of clinically invasive axillary lymph nodes (cN1) but also in the absence of suspicious lymph nodes (cN0) when the tumor is smaller than 2cm. An evaluation of overall survival and recurrence-free survival comparing patients with these sizes of tumors and adjusted for other prognostic factors should be performed to confirm these NAC indications.

## Supplementary Material

Supply

## Figures and Tables

**Table 1:** Characteristics of patients according to three periods of treatment Period 1 (P1) 2005-2009, period 2 (P2) 2010-2014, and period 3 (P3) 2015-2021.

		P1		P2		P3		Chi-2	Total	
		Nb	%	Nb	%	Nb	%	*p-value*	Nb	%
Age	median	48		50		50			50	
	CI 95%	48.0-50.6		49.7-51.9		50.8-52.6			50.4-51.6	
cT stage	cT0-1	19	6.8	42	8.3	179	21.2	**<0.0001**	240	14.7
	cT2	154	55	308	61.2	473	55.9		935	57.4
	cT3	82	29.3	132	26.2	136	16.1		350	21.5
	cT4	25	8.9	21	4.2	58	6.9		104	6.4
cN stage	cN0	121	43.2	196	39	473	55.9	**<0.0001**	790	48.5
	cN1	143	51.1	302	60	372	44		817	50.2
	cNx	16	5.7	5	1	1	0.1		22	1.4
Breast	Conservative	103	36.8	220	43.7	410	48.5	**0.003**	733	45
surgery	Mastectomy	177	63.2	283	56.3	436	51.5		896	55
Axillary	SLNB	5	1.8	82	16.3	265	31.3	**<0.0001**	352	21.6
surgery	ALND	208	74.3	305	60.6	488	57.7		1001	61.4
	SLNB+ALND	65	23.2	113	22.5	81	9.6		259	15.9
	No	2	0.7	3	0.6	12	1.4		17	1
Subtypes	Luminal A	101	36.1	138	27.4	154	18.2	**<0.0001**	393	24.1
	Luminal B Her2−	54	19.3	75	14.9	69	8.2		198	12.2
	Luminal Her2− Grade?	1	0.4	1	0.2	1	0.1		3	0.2
	Luminal B Her2+	42	15	82	16.3	178	21		302	18.5
	Her2+	30	10.7	63	12.5	127	15		220	13.5
	Triple Negative	52	18.6	144	28.6	317	37.5		513	31.5
ypT	ypT0	95	33.9	145	28.8	280	33.1	**0.043**	520	31.9
	ypTis	17	6.1	41	8.2	89	10.5		147	9
	ypT >= 1	168	60	317	63	477	56.4		962	59.1
pN	pN0sn	3	1.1	75	15	215	25.7	**<0.0001**	293	18.1
	pN1sn	2	0.7	8	1.6	53	6.3		63	3.9
	ypN0	167	60.1	200	40	302	36.1		669	41.4
	ypN1	106	38.1	217	43.4	267	31.9		590	36.5

Abbreviations: cT, clinical tumor size stage; cN, clinical lymph node status; SLNB, sentinel lymph node biopsy; ALND, axillary lymph node dissection; ypT, pathologic tumor status after neoadjuvant chemotherapy; pN pathologic lymph node status

**Table 2:** Axillary surgery for all patients and for clinically N0 patients.

		SLNB		ALND		SLNB + ALND		No Surgery		Chi-2
		Nb	%	Nb	%	Nb	%	Nb	%	*p-value*
**All patients**		352		1001		259		17		
Period	P1	5	1.4	208	20.8	65	25.1	2	11.8	<0.0001
	P2	82	23.3	305	30.5	113	43.6	3	17.6	
	P3	265	75.3	488	48.8	81	31.3	12	70.6	
Subtypes	Luminal A	44	12.5	273	27.3	73	28.2	3	17.6	<0.0001
	Luminal B Her2−	23	6.5	134	13.4	41	15.8	0	0	
	Luminal B Her2+	64	18.2	186	18.6	49	18.9	3	17.6	
	Her2+	45	12.8	147	14.7	25	9.7	3	17.6	
	Triple Negative	176	50	259	25.9	70	27	8	47.1	
	Luminal Her2− Grade?	0	0	2	0.2	1	0.4	0	0	
cN stage	cN0	328	93.2	282	28.2	168	64.9	12	70.6	<0.0001
	cN1	23	6.5	702	70.1	88	30.4	4	23.5	
	cNx	1	0.3	17	1.7	3	1.2	1	5.9	
cT stage	cT0-1	82	23.3	129	12.9	24	9.3	5	29.4	<0.0001
	cT2	254	72.2	481	48.1	190	73.9	8	47.1	
	cT3	16	4.5	291	29.1	40	15.6	3	17.6	
	cT4	0	0	100	10	3	1.2	1	5.9	
**only cN0 patients**		328		282		168		12		
Period	P1	5	1.5	69	24.5	46	27.4	1	8.3	<0.0001
	P2	76	23.2	54	19.1	65	38.7	1	8.3	
	P3	247	75.3	159	56.4	57	33.9	10	83.3	
Subtypes	Luminal A	43	13.1	90	31.9	46	27.4	2	16.7	<0.0001
	Luminal B Her2−	23	7	28	9.9	26	15.5	0	0	
	Luminal B Her2+	61	18.6	46	16.3	33	19.6	3	25	
	Her2+	44	13.4	46	16.3	16	9.5	1	8.3	
	Triple Negative	157	47.9	72	25.5	47	28	6	50	
cT stage	cT0-1	77	23.5	50	17.7	18	10.7	5	41.7	<0.0001
	cT2	237	72.3	137	48.6	125	74.4	6	50	
	cT3	14	4.3	81	28.7	24	14.3	1	8.3	
	cT4	0	0	14	5	1	0.6	0	0	

Abbreviations: cT, clinical tumor size stage; cN, clinical lymph node status; SLNB, sentinel lymph node biopsy; ALND, axillary lymph node dissection

**Table 3:** Results of regression analysis to determine associations between periods, tumor subtypes, and clinical tumor size for SLNB versus ALND, and clinical lymph node status for cT2-3-4 versus cT0-1, and clinical tumor size and clinical lymph node status for mastectomy versus breast conservative surgery.

			CI 95%	
	*p-value*	OR	Inferior	Superior
**SLNB *versus* ALND**				
Luminal A		1		
Luminal B Her2−	0.206	1.462	0.811	2.634
Luminal B Her2+	0.053	1.631	0.994	2.677
Her2+	0.355	1.286	0.754	2.193
Triple Negative	**<0.0001**	**2.332**	**1.511**	**3.597**
cT0-1		1		
cT2	0.086	1.431	0.95	2.154
cT3	**<0.0001**	**0.254**	**0.147**	**0.44**
cT4	**0.003**	**0.041**	**0.005**	**0.329**
P1		1		
P2	**<0.0001**	**3.567**	**2.12**	**6.001**
P3	**0.008**	**1.877**	**1.181**	**2.983**
**cT2-3-4 *versus* cT0-1**				
P1		1		
P2	0.371	0.771	0.435	1.365
P3	**<0.0001**	**0.27**	0.162	0.45
Luminal A		1		
Lum B Her2−	0.981	1.006	0.606	1.671
Lum B Her2+	0.385	1.211	0.786	1.868
Her2+	0.062	1.617	0.976	2.681
Triple Neg	0.163	1.315	0.895	1.933
cN0		1		
cN>=1	**<0.0001**	**1.748**	1.306	2.34
**mastectomy *versus* BCS**				
P1		1		
P2	0.224	0.818	0.591	1.131
P3	0.114	0.778	0.57	1.062
cT0-1		1		
cT2	0.381	0.877	0.653	1.177
cT3	**<0.0001**	**2.974**	2.058	4.298
cT4	**<0.0001**	**6.469**	3.324	12.589
Luminal A		1		
Lum B Her2−	0.085	0.725	0.503	1.045
Lum B Her2+	0.662	0.93	0.673	1.287
Her2+	0.259	1.234	0.856	1.779
Triple Neg	**<0.0001**	**0.567**	0.426	0.756
cN0		1		
cN1	**<0.0001**	**1.518**	1.223	1.883
cNx	0.684	1.223	0.464	3.222

Abbreviations: cT, clinical tumor size stage; cN, clinical lymph node status; SLNB, sentinel lymph node biopsy; ALND, axillary lymph node dissection; BCS, breast conservative surgery.

**Table 4:** Characteristics of patients according to clinical tumor size, cT0-1 versus cT2-3-4.

		cT0-1		cT2-3-4		Chi-2
		Nb	%	Nb	%	*p-value*
**All patients**		240	14.7	1389	85.3	
Period	P1	19	7.9	261	18.8	**<0.0001**
	P2	42	17.5	461	33.2	
	P3	179	74.6	667	48	
Subtypes	Luminal A	58	24.2	335	24.1	0.154
	Luminal B Her2−	27	11.2	171	12.3	
	Luminal Her2+ G?	2	0.8	1	0.1	
	Luminal B Her2+	47	19.6	255	18.4	
	Her2+	27	11.2	193	13.9	
	Triple Negative	79	32.9	434	31.2	
cN stage	cN0	150	62.5	640	46.1	**<0.0001**
	cN1	90	37.5	727	52.3	
	cNx	0	0	22	1.6	
**Luminal A**		58	14.8	335	85.2	
Period	P1	6	10.3	95	28.4	**<0.0001**
	P2	14	24.1	124	37	
	P3	38	65.5	116	34.6	
cN stage	cN0	35	60.3	146	43.6	**0.041**
	cN1	23	39.7	181	54	
	cNx	0	0	8	2.4	
**Luminal B Her2−**		27	13.6	171	86.4	
Period	P1	6	22.2	48	28.1	0.295
	P2	8	29.6	67	39.2	
	P3	13	48.1	56	32.7	
cN stage	cN0	15	55.6	62	36.3	0.135
	cN1	12	44.4	105	61.4	
	cNx	0	0	4	2.3	
**Luminal B Her2+ & Her2+**		74	14.2	448	85.8	
Period	P1	3	4.1	69	15.4	**<0.0001**
	P2	6	8.1	139	31	
	P3	65	87.8	240	53.6	
cN stage	cN0	45	60.8	205	45.8	0.051
	cN1	29	39.2	241	53.8	
	cNx	0	0	2	0.4	
**Triple Negative**		79	15.4	434	84.6	
Period	P1	3	3.8	49	11.3	**0.003**
	P2	14	17.7	130	30	
	P3	62	78.5	255	58.8	
cN stage	cN0	55	69.6	227	52.3	**0.012**
	cN1	24	30.4	199	45.9	
	cNx	0	0	8	1.8	

Abbreviations: cN, clinical lymph node status.

**Table 5:** Factors associated with residual breast tumor and pathologic complete response (pCR) in univariate analysis.

		ypT0-is		ypT≥1		Chi-2
		Nb	%	Nb	%	*p-value*
All patients		664	40.8	965	59.2	
Period	P1	112	16.9	168	17.4	0.073
	P2	186	28	317	32.8	
	P3	366	55.1	480	49.7	
Subtypes	Luminal A	56	8.4	337	34.9	**<0.0001**
	Luminal B Her2−	57	8.6	141	14.6	
	Luminal B Her2+	148	22.3	154	16	
	Her2+	149	22.4	71	7.4	
	Triple Negative	252	38	261	27	
	Luminal Her2− Grade?	2	0.3	1	0.1	
cT stage	cT0-1	109	16.4	131	13.6	0.087
	cT2	390	58.7	545	56.5	
	cT3	130	19.6	220	22.8	
	cT4	35	5.3	69	7.2	
cN stage	cN0	322	48.5	468	48.5	1
	cN1	333	50.2	484	50.2	
	cNx	9	1.4	13	1.3	
	Luminal A Grade 1	2	3.6	69	20.5	**0.001**
	Luminal A Grade 2	54	96.4	268	79.5	
		pCR		no pCR		Chi-2
		Nb	%	Nb	%	*p-value*
All patients		561	34.4	1068	65.6	
Period	P1	94	16.8	186	17.4	**0.011**
	P2	149	26.6	354	33.1	
	P3	318	56.7	528	49.4	
Subtypes	Luminal A	30	5.3	363	34	**<0.0001**
	Luminal B Her2−	41	7.3	157	14.7	
	Luminal B Her2+	128	22.8	174	16.3	
	Her2+	138	24.6	82	7.7	
	Triple Negative	223	39.8	290	27.2	
	Luminal Her2− Grade?	1	0.2	2	0.2	
cT stage	cT0-1	89	15.9	151	14.1	**0.045**
	cT2	340	60.6	595	55.7	
	cT3	102	18.2	248	23.2	
	cT4	30	5.3	74	6.9	
cN stage	cN0	311	55.4	479	44.9	**<0.0001**
	cN1	243	43.3	574	53.7	
	cNx	7	1.2	15	1.4	
	Luminal A Grade 1	2	6.7	69	19	0.065
	Luminal A Grade 2	28	93.3	294	81	

Abbreviations: ypT, pathologic tumor status after NAC; cT, clinical tumor size stage; cN, clinical lymph node status.

**Table 6:** Regression analysis to determine factors significantly associated with residual breast tumor and pathologic complete response (pCR).

**residual tumor *versus* ypT0-is**		*p-value*	OR	CI 95%	
				Inferior	Superior
cT stage	cT2-3-4		1		
	cT0-1	0.079	0.766	0.569	1.031
Subtypes	Luminal A		**1**		
	Lum B Her2−	**<0,0001**	**0.409**	0.269	0.622
	Lum B Her2+	**<0,0001**	**0.173**	0.12	0.248
	Her2+	**<0,0001**	**0.078**	0.052	0.117
	Triple Neg	**<0,0001**	**0.172**	0.123	0.239
no pCR *versus* pCR		*p-value*	OR	CI 95%	
				Inferior	Superior
cT stage	cT2-3-4		1		
	cT0-1	0.447	0.885	0.647	1.212
Subtypes	Luminal A		1		
	Lum B Her2−	**<0,0001**	**0.305**	0.183	0.507
	Lum B Her2+	**<0,0001**	**0.11**	0.071	0.171
	Her2+	**<0,0001**	**0.048**	0.03	0.076
	Triple Neg	**<0,0001**	**0.109**	0.072	0.165
cN stage	cN0		1		
	cN1	**<0,0001**	**1.499**	1.194	1.882
	cNx	0.845	0.905	0.332	2.468

Abbreviations: ypT, pathologic tumor status after NAC; cT, clinical tumor size stage; cN, clinical lymph node status.

**Table 7:** Pathologic axillary nodal status.

	Luminal A		Lum B Her2−		Lum B Her2+		Her2+		Triple Neg		Luminal Her2− Grade?		Total	
	Nb	%	Nb	%	Nb	%	Nb	%	Nb	%	Nb	%	Nb	%
**cN0**														
pN0sn	29	16.1	20	26	53	37.9	35	33	138	50	0	0	275	35.3
pN1sn	15	8.3	4	5.2	9	6.4	8	7.5	19	6.9	0	0	55	7.1
ypN0	71	39.4	33	42.9	68	48.6	52	49.1	91	33	0	0	315	40.4
ypN1	65	36.1	20	26	10	7.1	11	10.4	28	10.1	0	0	134	17.2
**cN1**														
pN0sn	0	0	0	0	3	1.9	1	0.9	12	5.5	0	0	16	2
pN1sn	1	0.5	1	0.9	0	0	0	0	5	2.3	0	0	7	0.9
ypN0	37	18.3	41	35	87	55.1	74	66.7	104	47.7	1	33.3	344	42.5
ypN1	164	81.2	75	64.1	68	43	36	32.4	97	44.5	2	66.7	442	54.6

Abbreviations: cN, clinical lymph node status; ypN, pathologic lymph node status after NAC

**Table 8:** Significant associations between breast conservative surgery or mastectomy in univariate analysis.

		Conservative		Mastectomy		Chi-2
		Nb	%	Nb	%	*p-value*
All patients		733	45	896	55	
Period	P1	103	14.1	177	19.8	**0.002**
	P2	220	30	283	31.6	
	P3	410	55.9	436	48.7	
Subtypes	Luminal A	151	20.6	242	27	**<0.0001**
	Luminal B Her2−	90	12.3	108	12.1	
	Luminal B Her2+	129	17.6	173	19.3	
	Her2+	76	10.4	144	16.1	
	Triple Negative	285	38.9	228	25.4	
	Luminal Her2− Grade?	2	0.3	1	0.1	
cT stage	cT0-1	127	17.3	113	12.6	**<0.0001**
	cT2	509	69.4	426	47.5	
	cT3	85	11.6	265	29.6	
	cT4	12	1.6	92	10.3	
cN stage	cN0	423	57.7	367	41	**<0.0001**
	cN1	302	41.2	515	57.4	
	cNx	8	1.1	14	1.6	
ypT	ypT0	246	33.6	274	30.6	0.274
	ypTis	70	9.5	77	8.6	
	ypT >= 1	417	56.9	545	60.8	
pCR	Yes	273	37.2	288	32.1	**0.018**
	No	460	62.8	608	67.9	

Abbreviations: cT, clinical tumor size stage; cN, clinical lymph node status; ypT, pathologic tumor status after neoadjuvant chemotherapy; pCR, pathologic complete response.

## Data Availability

Not applicable.

## Abbreviations:

NAC: neoadjuvant chemotherapy
pCR: pathological complete response
TN: triple negative
BC: breast cancer
SLNB: sentinel lymph node biopsy
ALND: axillary lymph node dissection
ER: Endocrine Receptor

## References

[1] Bonadonna G Karnofsky memorial lecture. Conceptual and practical advances in the management of breast cancer. J Clin Oncol 7 (1989): 1380–1397. 2674330 10.1200/JCO.1989.7.10.1380

[2] Rubens RD, Sexton S, Tong D, Winter PJ, Knight RK, Hayward JL. Combined chemotherapy and radiotherapy for locally advanced breast cancer. Eur J Cancer 16 (1965): 351–356. 10.1016/0014-2964(80)90352-7 7371690

[3] Kaufmann M, Von Minckwitz G, Bear HD, Recommendations from an international expert panel on the use of neoadjuvant (primary) systemic treatment of operable breast cancer: new perspectives 2006. Ann Oncol 18 (2007): 1927–1934. 17998286 10.1093/annonc/mdm201

[4] Asselain B, Barlow W, Bartlett J, Long-term outcomes for neoadjuvant versus adjuvant chemotherapy in early breast cancer: meta-analysis of individual patient data from ten randomised trials. Lancet Oncol 19 (2018): 27–39. 29242041 10.1016/S1470-2045(17)30777-5 PMC5757427

[5] Clough KB, Gouveia PF, Benyahi D, Positive Margins After Oncoplastic Surgery for Breast Cancer. Ann Surg Oncol 22 (2015): 4247–4253. 25893409 10.1245/s10434-015-4514-3

[6] Houvenaeghel G, Lambaudie E, Bannier M, Positive or close margins: reoperation rate and second conservative resection or total mastectomy? Cancer Manag Res 11 (2019): 2507–2516. 30992681 10.2147/CMAR.S190852 PMC6445211

[7] Early Breast Cancer Trialists’ Collaborative Group (EBCTCG). Long-term outcomes for neoadjuvant versus adjuvant chemotherapy in early breast cancer: meta-analysis of individual patient data from ten randomised trials. Lancet Oncol 19 (2018): 27–39. 29242041 10.1016/S1470-2045(17)30777-5 PMC5757427

[8] Sorlie T, Perou CM, Tibshirani R, Gene expression patterns of breast carcinomas distinguish tumor subclasses with clinical implications. Proc Natl Acad Sci 98 (2001): 10869–10874. 11553815 10.1073/pnas.191367098 PMC58566

[9] Rouzier R, Perou CM, Symmans WF, Breast Cancer Molecular Subtypes Respond Differently to Preoperative Chemotherapy. Clin Cancer Res 11 (2005): 5678–585. 16115903 10.1158/1078-0432.CCR-04-2421

[10] Cortazar P, Zhang L, Untch M, Pathological complete response and long-term clinical benefit in breast cancer: the CTNeoBC pooled analysis. The Lancet 384 (2014): 164–172. 10.1016/S0140-6736(13)62422-8 24529560

[11] Broglio KR, Quintana M, Foster M, Association of Pathologic Complete Response to Neoadjuvant Therapy in HER2-Positive Breast Cancer With Long-Term Outcomes: A Meta-Analysis. JAMA Oncol 2 (2016): 751. 26914222 10.1001/jamaoncol.2015.6113

[12] Huang M, O’Shaughnessy J, Zhao J, Association of Pathologic Complete Response with Long-Term Survival Outcomes in Triple-Negative Breast Cancer: A Meta-Analysis. Cancer Res 80 (2020): 5427–5434. 32928917 10.1158/0008-5472.CAN-20-1792

[13] Von Minckwitz G, Huang CS, Mano MS, Trastuzumab Emtansine for Residual Invasive HER2-Positive Breast Cancer. N Engl J Med 380 (2019): 617–628. 30516102 10.1056/NEJMoa1814017

[14] Masuda N, Lee SJ, Ohtani S, Adjuvant Capecitabine for Breast Cancer after Preoperative Chemotherapy. N Engl J Med 376 (2017): 2147–2159. 28564564 10.1056/NEJMoa1612645

[15] Bear HD, Anderson S, Smith RE, Sequential preoperative or postoperative docetaxel added to preoperative doxorubicin plus cyclophosphamide for operable breast cancer:National Surgical Adjuvant Breast and Bowel Project Protocol B-27. J Clin Oncol Off J Am Soc Clin Oncol 24 (2006): 2019–2027. 10.1200/JCO.2005.04.1665 16606972

[16] Von Minckwitz G, Untch M, Blohmer JU, Definition and Impact of Pathologic Complete Response on Prognosis After Neoadjuvant Chemotherapy in Various Intrinsic Breast Cancer Subtypes. J Clin Oncol 30 (2012): 1796–1804. 22508812 10.1200/JCO.2011.38.8595

[17] Agha R, Abdall-Razak A, Crossley E, The STROCSS 2019 Guideline: Strengthening the Reporting of Cohort Studies in Surgery. International Journal of Surgery 72 (2019): 156–165. 31704426 10.1016/j.ijsu.2019.11.002

[18] Penault-Llorca F, Balaton A, Sabourin JC, Immunochemistry evaluation of HER2 status in infiltration breast cancer: technical protocol and interpretation guidelines. Ann Pathol 22 (2002): 150–157. 12124503

[19] Cardoso F, Kyriakides S, Ohno S, Early breast cancer: ESMO Clinical Practice Guidelines for diagnosis, treatment and follow-up†. Ann Oncol 30 (2019): 1194–220. 31161190 10.1093/annonc/mdz173

[20] Romond EH, Perez EA, Bryant J, Trastuzumab plus adjuvant chemotherapy for operable HER2-positive breast cancer. N Engl J Med 353 (2005): 1673–1684. 16236738 10.1056/NEJMoa052122

[21] Piccart-Gebhart MJ, Procter M, Leyland-Jones B, Trastuzumab after adjuvant chemotherapy in HER2-positive breast cancer. N Engl J Med 353 (2005): 1659–1672. 16236737 10.1056/NEJMoa052306

[22] Slamon D, Eiermann W, Robert N, Adjuvant Trastuzumab in HER2-Positive Breast Cancer. N Engl J Med 365 (2011): 1273–1283. 21991949 10.1056/NEJMoa0910383 PMC3268553

[23] Joensuu H, Kellokumpu-Lehtinen PL, Bono P, Adjuvant Docetaxel or Vinorelbine with or without Trastuzumab for Breast Cancer. N Engl J Med 354 (2006): 809–820. 16495393 10.1056/NEJMoa053028

[24] De Nonneville A, Gonçalves A, Zemmour C, Benefit of adjuvant chemotherapy with or without trastuzumab in pT1ab node-negative human epidermal growth factor receptor 2-positive breast carcinomas: results of a national multi-institutional study. Breast Cancer Res Treat (2017). 10.1007/s10549-017-4136-5 28155054

[25] Tolaney SM, Barry WT, Dang CT, Adjuvant Paclitaxel and Trastuzumab for Node-Negative, HER2-Positive Breast Cancer. N Engl J Med 372 (2015): 134–141. 25564897 10.1056/NEJMoa1406281 PMC4313867

[26] De Nonneville A, Gonçalves A, Zemmour C, Adjuvant chemotherapy in pT1ab node-negative triple-negative breast carcinomas: Results of a national multi-institutional retrospective study. Eur J Cancer 84 (2017): 34–43. 28780480 10.1016/j.ejca.2017.06.043

[27] An X, Lei X, Huang R, Adjuvant chemotherapy for small, lymph node- negative, triple-negative breast cancer: A single-center study and a meta-analysis of the published literature. Cancer 126 (2020): 3837–3846. 32710666 10.1002/cncr.32878

[28] Lluch A, Barrios CH, Torrecillas L, Phase III Trial of Adjuvant Capecitabine After Standard Neo-/Adjuvant Chemotherapy in Patients With Early Triple-Negative Breast Cancer (GEICAM/2003-11_CIBOMA/2004-01). J Clin Oncol Off J Am Soc Clin Oncol 38 (2020): 203–213. 10.1200/JCO.19.00904 PMC6968797 31804894

[29] Joensuu H, Kellokumpu-Lehtinen PL, Huovinen R, Adjuvant Capecitabine in Combination With Docetaxel, Epirubicin, and Cyclophosphamide for Early Breast Cancer. JAMA Oncol 3 (2017): 793–800. 28253390 10.1001/jamaoncol.2016.6120 PMC5824321

[30] Martín M, Ruiz Simón A, Ruiz Borrego M, Epirubicin Plus Cyclophosphamide Followed by Docetaxel Versus Epirubicin Plus Docetaxel Followed by Capecitabine As Adjuvant Therapy for Node-Positive Early Breast Cancer: Results From the GEICAM/2003-10 Study. J Clin Oncol Off J Am Soc Clin Oncol 33 (2015): 3788–3795. 10.1200/JCO.2015.61.9510 26416999

[31] O’Shaughnessy J, Koeppen H, Xiao Y, Patients with Slowly Proliferative Early Breast Cancer Have Low Five-Year Recurrence Rates in a Phase III Adjuvant Trial of Capecitabine. Clin Cancer Res Off J Am Assoc Cancer Res 21 (2015): 4305–4311. 10.1158/1078-0432.CCR-15-0636 26041745

[32] Von Minckwitz G, Conrad B, Reimer T, Decker T, A randomized phase 2 study comparing EC or CMF versus nab-paclitaxel plus cape-citabine as adjuvant chemotherapy for nonfrail elderly patients with moderate to high-risk early breast cancer (ICE II-GBG 52). Cancer 121 (2015): 3639–3648. 26111104 10.1002/cncr.29506

[33] Ohno S, Chow LWC, Sato N, Randomized trial of preoperative docetaxel with or without capecitabine after 4 cycles of 5-fluorouracil- epirubicin- cyclophosphamide (FEC) in early-stage breast cancer: exploratory analyses identify Ki67 as a predictive biomarker for response to neoadjuvant chemotherapy. Breast Cancer Res Treat 142 (2013): 69–80. 24122389 10.1007/s10549-013-2691-y PMC3825616

[34] Von Minckwitz G, Rezai M, Fasching PA, Survival after adding capecitabine and trastuzumab to neoadjuvant anthracy-cline-taxane-based chemotherapy for primary breast cancer (GBG 40--GeparQuattro). Ann Oncol Off J Eur Soc Med Oncol 25 (2014): 81–89. 10.1093/annonc/mdt410 24273046

[35] Von Minckwitz G, Blohmer JU, Costa SD, Response-Guided Neoadjuvant Chemotherapy for Breast Cancer. J Clin Oncol 31 (2013): 3623–3630. 24002511 10.1200/JCO.2012.45.0940

[36] Xu D, Chen X, Li X, Addition of Capecitabine in Breast Cancer First-line Chemotherapy Improves Survival of Breast Cancer Patients. J Cancer 10 (2019): 418–429. 30719136 10.7150/jca.29739 PMC6360291

[37] Natori A, Ethier JL, Amir E, Capecitabine in early breast cancer: A meta-analysis of randomised controlled trials. Eur J Cancer Oxf Engl 77 (2017): 40–47. 10.1016/j.ejca.2017.02.024 28355581

[38] Tutt ANJ, Garber JE, Kaufman B, Adjuvant Olaparib for Patients with BRCA1- or BRCA2-Mutated Breast Cancer. N Engl J Med 384 (2021): 2394–2405. 34081848 10.1056/NEJMoa2105215 PMC9126186

[39] Dauplat J, Kwiatkowski F, Rouanet P, Quality of life after mastectomy with or without immediate breast reconstruction. Br J Surg 104 (2017): 1197–1206. 28401542 10.1002/bjs.10537

[40] Quilichini O, Barrou J, Bannier M, Mastectomy with immediate breast reconstruction: Results of a mono-centric 4-years cohort. Ann Med Surg 61 (2012):172–179. 10.1016/j.amsu.2020.12.033 PMC7787913 33437474

[41] Kuehn T, Bauerfeind I, Fehm T, Senti-nel-lymph-node biopsy in patients with breast cancer before and after neoadjuvant chemotherapy (SENTINA): a prospective, multicentre cohort study. Lancet Oncol 14 (2013): 609–618. 23683750 10.1016/S1470-2045(13)70166-9

[42] El Hage Chehade H, Headon H, El Tokhy O, Is sentinel lymph node biopsy a viable alternative to complete axillary dissection following neoadjuvant chemotherapy in women with node-positive breast cancer at diagnosis? An updated meta-analysis involving 3,398 patients. Am J Surg 212 (2016): 969–981. 27671032 10.1016/j.amjsurg.2016.07.018

[43] Galimberti V, Ribeiro FSK, Maisonneuve P, Sentinel node biopsy after neoadjuvant treatment in breast cancer: Five-year follow-up of patients with clinically node-negative or node-positive disease before treatment. Eur J Surg Oncol J Eur Soc Surg Oncol Br Assoc Surg Oncol 42 (2016): 361–368. 10.1016/j.ejso.2015.11.019 26746091

[44] Menard JP, Extra JM, Jacquemier J, Sentinel lymphadenectomy for the staging of clinical axillary node-negative breast cancer before neoadjuvant chemotherapy. Eur J Surg Oncol 35 (2009): 916–920. 19157769 10.1016/j.ejso.2008.11.002

[45] Krag DN, Anderson SJ, Julian TB, Sentinel-lymph-node resection compared with conventional axillary-lymph-node dissection in clinically node-negative patients with breast cancer: overall survival findings from the NSABP B-32 randomised phase 3 trial. Lancet Oncol 11 (2010): 927–933. 20863759 10.1016/S1470-2045(10)70207-2 PMC3041644

[46] Houvenaeghel G, De Nonneville A, Cohen M, Lack of prognostic impact of sentinel node micro-metastases in endocrine receptor-positive early breast cancer: results from a large multicenter cohort. ESMO Open 6 (2021): 100–151. 10.1016/j.esmoop.2021.100151 PMC8314870 33984674

[47] Houvenaeghel G, Sabatier R, Reyal F, Axillary lymph node micrometastases decrease triple-negative early breast cancer survival. Br J Cancer 115 (2016): 1024–1031. 27685443 10.1038/bjc.2016.283 PMC5117781

[48] Boughey JC, Suman VJ, Mittendorf EA, Ahrendt GM, Sentinel lymph node surgery after neoadjuvant chemotherapy in patients with node-positive breast cancer: the ACOSOG Z1071 (Alliance) clinical trial. JAMA 310 (2013): 1455–1461. 24101169 10.1001/jama.2013.278932 PMC4075763

[49] Classe JM, Loaec C, Gimbergues P, Sentinel lymph node biopsy without axillary lymphadenectomy after neoadjuvant chemotherapy is accurate and safe for selected patients: the GANEA 2 study. Breast Cancer Res Treat 173 (2019): 343–352. 30343457 10.1007/s10549-018-5004-7

[50] Caudle AS, Yang WT, Krishnamurthy S, Improved Axillary Evaluation Following Neoadjuvant Therapy for Patients With Node-Positive Breast Cancer Using Selective Evaluation of Clipped Nodes: Implementation of Targeted Axillary Dissection. J Clin Oncol Off J Am Soc Clin Oncol 34 (2016): 1072–1078. 10.1200/JCO.2015.64.0094 PMC4933133 26811528

[51] Donker M, Straver ME, Wesseling J, Marking axillary lymph nodes with radioactive iodine seeds for axillary staging after neoadjuvant systemic treatment in breast cancer patients: the MARI procedure. Ann Surg 261 (2015): 378–382. 24743607 10.1097/SLA.0000000000000558

[52] Samiei S, Simons JM, Engelen SME, Axillary Pathologic Complete Response After Neoadjuvant Systemic Therapy by Breast Cancer Subtype in Patients With Initially Clinically Node-Positive Disease: A Systematic Review and Meta-analysis. JAMA Surg 156 (2021): e210891. 33881478 10.1001/jamasurg.2021.0891 PMC8060891

