# Supplementary material for: Neoadjuvant Chemotherapy for Breast Cancer: Evolution of Clinical Practice in a French Cancer Center Over 16 Years and Pathologic Response Rates According to Tumor Subtypes and Clinical Tumor Size: Retrospective Cohort Study
Source: J Surg Res (Houst). Author manuscript; Available in PMC 2023 Jan 26. (PMC9879000; doi:10.26502/jsr.10020251)
Supplement: Supply [file NIHMS1856659-supplement-Supply.docx]

|  |  |  | **P1** |  | **P2** |  | **P3** |  | Ch-2 | Chi-2 § |
| --- | --- | --- | --- | --- | --- | --- | --- | --- | --- | --- |
|  |  |  | Nb | % | Nb | % | Nb | % | *p-value* | *p-value* |
| Luminal A | cN0 | cT0-1 | 4 | 7.7 | 3 | 5.7 | 28* | 36.8 | **<0.0001** | **<0.0001** |
|  |  | cT2-3-4 | 48 | 92.3 | 50 | 94.3 | 48 | 63.2 |  |  |
|  | cN1 | cT0-1 | 2 | 4.8 | 11 | 13.1 | 10° | 12.8 | 0.325 |  |
|  |  | cT2-3-4 | 40 | 95.2 | 73 | 86.9 | 68° | 87.2 |  |  |
| Lum B Her2- | cN0 | cT0-1 | 2 | 11.1 | 4 | 13.8 | 9 | 30 | 0.172 | **0.02** |
|  |  | cT2-3-4 | 16 | 88.9 | 25 | 86.2 | 21 | 70 |  |  |
|  | cN1 | cT0-1 | 4 | 12.5 | 4 | 8.7 | 4 | 10.3 | 0.862 |  |
|  |  | cT2-3-4 | 28 | 87.5 | 42 | 91.3 | 35 | 89.7 |  |  |
| Lum B Her2+ | cN0 | cT0-1 | 1 | 7.1 | 1 | 3 | 24 | 25 | **0.01** | **0.008** |
|  |  | cT2-3-4 | 13 | 92.9 | 32 | 97 | 72 | 75 |  |  |
|  | cN1 | cT0-1 | 1 | 3.7 | 3 | 6.1 | 17 | 20.7 | **0.016** |  |
|  |  | cT2-3-4 | 26 | 96.3 | 46 | 93.9 | 65 | 79.3 |  |  |
| Her2+ | cN0 | cT0-1 | 0 | 0 | 0 | 0 | 19 | 25.7 | **0.006** | **0.009** |
|  |  | cT2-3-4 | 12 | 100 | 21 | 100 | 55 | 74.3 |  |  |
|  | cN1 | cT0-1 | 1 | 5.6 | 2 | 4.9 | 5 | 9.4 | 0.669 |  |
|  |  | cT2-3-4 | 17 | 94.4 | 39 | 95.1 | 48 | 90.6 |  |  |
| Triple Negative | cN0 | cT0-1 | 0 | 0 | 5 | 8.3 | 50 | 25.4 | **0.001** | **<0.0001** |
|  |  | cT2-3-4 | 25 | 100 | 55 | 91.7 | 147 | 74.6 |  |  |
|  | cN1 | cT0-1 | 3 | 13 | 9 | 11.1 | 12 | 10.1 | 0.909 |  |
|  |  | cT2-3-4 | 20 | 87 | 72 | 88.9 | 107 | 89.9 |  |  |

**Supplementary table 1:** Distribution of patients for the three periods according to tumor subtypes and cN status. §: cN0 *versus* cN1. Abbreviations: cN, clinical lymph node status.

|  |  | **ypT0-is** |  | **ypT≥1** |  | **Chi-2** |
| --- | --- | --- | --- | --- | --- | --- |
|  |  | Nb | % | Nb | % | *p-value* |
| Luminal A | | 56 | 14.4 | 334 | 85.6 |  |
| pN status | pN0sn | 4 | 7.1 | 25 | 7.5 | **0.005** |
|  | pN1sn | 0 | 0 | 16 | 3.6 |  |
|  | ypN0 | 26 | 46.4 | 83 | 25.4 |  |
|  | ypN1 | 26 | 46.4 | 210 | 62.9 |  |
| Luminal B Her2- | | 57 | 28.8 | 141 | 71.2 |  |
| pN status | pN0sn | 5 | 8.8 | 15 | 10.6 | **<0.0001** |
|  | pN1sn | 0 | 0 | 5 | 3.5 |  |
|  | ypN0 | 36 | 63.2 | 40 | 28.4 |  |
|  | ypN1 | 16 | 28.1 | 81 | 57.4 |  |
| Luminal B Her2+ | | 147 | 49.2 | 152 | 50.8 |  |
| pN status | pN0sn | 26 | 17.7 | 30 | 19.7 | **<0.0001** |
|  | pN1sn | 5 | 3.4 | 4 | 2.6 |  |
|  | ypN0 | 96 | 65.3 | 59 | 38.8 |  |
|  | ypN1 | 20 | 13.6 | 59 | 38.8 |  |
| Her2+ ER- | | 146 | 67.3 | 71 | 32.7 |  |
| pN status | pN0sn | 25 | 17.1 | 11 | 15.5 | **<0.0001** |
|  | pN1sn | 6 | 4.1 | 2 | 2.8 |  |
|  | ypN0 | 104 | 71.2 | 22 | 31 |  |
|  | ypN1 | 11 | 7.5 | 36 | 50.7 |  |
| Triple negative | | 248 | 49.4 | 254 | 50.6 |  |
| pN status | pN0sn | 77 | 31 | 74 | 29.1 | **<0.0001** |
|  | pN1sn | 13 | 5.2 | 11 | 4.3 |  |
|  | ypN0 | 129 | 52 | 71 | 28 |  |
|  | ypN1 | 29 | 11.7 | 98 | 38.6 |  |

**Supplementary table 2:** Breast pathologic complete response according to tumor subtypes and axillary nodal status. Luminal A: 26/52 ypN1: 50.0%, Luminal B Her2-negative: 16/52 ypN1: 30.8%, Luminal B Her2-positive: 20/116 ypN1: 17.2%, Her2-positive ER-negative: 11/115 ypN1: 9.6%, Triple negative: 29/158 ypN1: 18.35%. Abbreviations: pN pathologic lymph node status
